# Silver nanoparticle loaded collagen/chitosan scaffolds promote wound healing via regulating fibroblast migration and macrophage activation

**DOI:** 10.1038/s41598-017-10481-0

**Published:** 2017-09-05

**Authors:** Chuangang You, Qiong Li, Xingang Wang, Pan Wu, Jon Kee Ho, Ronghua Jin, Liping Zhang, Huawei Shao, Chunmao Han

**Affiliations:** Department of Burns & Wound Care Center, Second Affiliated Hospital of Medical College, Zhejiang University, Hangzhou, 310009 China

## Abstract

Treatment of full-thickness skin defects poses significant clinical challenges including risk of infection and severe scaring. Silver nanoparticle (NAg), an effective antimicrobial agent, has provided a promising therapeutic method for burn wounds. However, the detailed mechanism remains unknown. Hence, we constructed a metallic nanosilver particles-collagen/chitosan hybrid scaffold (NAg-CCS) and investigated its potential effects on wound healing. *In vitro* scratch assay, immunofluorescence staining and antibacterial activity of the scaffold were all studied. *In vivo* NAg-CCS was applied in full-thickness skin defects in Sprague-Dawley (SD) rats and the therapeutic effects of treatment were evaluated. The results showed that NAg at a concentration of 10 ppm accelerated the migration of fibroblasts with an increase in expression of α-smooth muscle actin (α-SMA). Furthermore, *in vivo* studies showed increased levels of pro-inflammatory and scar-related factors as well as α-SMA, while markers for macrophage activation were up-regulated. On day 60 post transplantation of ultra-thin skin graft, the regenerated skin by NAg-CCS had a similar structure to normal skin. In summary, we demonstrated that NAg-CCS was bactericidal, anti-inflammatory and promoted wound healing potentially by regulating fibroblast migration and macrophage activation, making it an ideal dermal substitute for wound regeneration.

## Introduction

Skin is the largest and one of the most complex organs in the human body, easily affected by various harmful external factors^1, 2^. Acute or chronic wounds represent a major unresolved clinical problem. At present, the main treatment strategy for full-thickness skin defects is to ensure rapid wound closure by cell therapy and/or skin transplantation^3^. However, the absence or severe damage of dermis prevents the epithelialization of wounds, indicating that dermal reconstruction is a critical procedure in wound healing^4^. Tissue engineering method could be a good way for dermal repair and regeneration. To date, many tissue-engineered products have been used for skin defects. An ideal dermal substitute should maintain a moist environment at the wound interface, allow gaseous exchange, act as a barrier against microorganisms, and remove excess exudates^5^. The substitute should also be non-toxic, non-allergenic and non-adherent in the sense that the product can be easily removed without trauma, and made from a readily available biomaterial that requires minimal processing, possesses antimicrobial properties, and promotes wound healing^6^.

Well-designed scaffold with a suitable porous structure can support cell migration and guide vascular infiltration, making it an ideal dermal substitute for wound regeneration. Several *in vitro* studies that focused on determining the optimal mean pore size of collagen-based scaffolds indicated that large pores (≥250 µm) favor cell attachment, proliferation and migration^7–9^. A few products have already been put into market and applied to clinical use. However, these often show difficulty in resisting infection and inflammation. Generally, skin defects with a large amount of fluid loss and bacterial infection, such as severe burns, may lead to serious consequences. In addition, exudates between the wound and dressing serve as a trigger of infection and acute inflammatory cells, which could prevent the wound from healing^10^. Wound dressings with antibacterial agents have been regarded as one of the most suitable approaches for treating wound infection.

Collagen-based dermal scaffolds coated with NAg could be a new type of antimicrobial dressing with non-toxic components. NAg can destroy the membrane easily, pass into the microbial body and convert into silver ions (Ag^+^) in the cytoplasm with damaging the intracellular structure as a secondary result^11^. NAg undergoes a shape-dependent interaction with the gram-negative bacterium *Escherichia Coli (E. Coli)*. Hence, it has been speculated that NAg with the same surface area but with different shapes may also have different effective surface areas^12^. These composite scaffolds coated with NAg were bactericidal and exhibited efficient blood clotting capabilities^13, 14^. Cell attachment studies have shown that cells were well-attached to the scaffold, suggesting its potential as a material for wound dressings. The incorporation of silver into alginate fibers has also been shown to increase antimicrobial activity and improve the binding affinity to elastase, matrix metalloprotease-2 and pro-inflammatory cytokines^15^.

**Figure 1:**
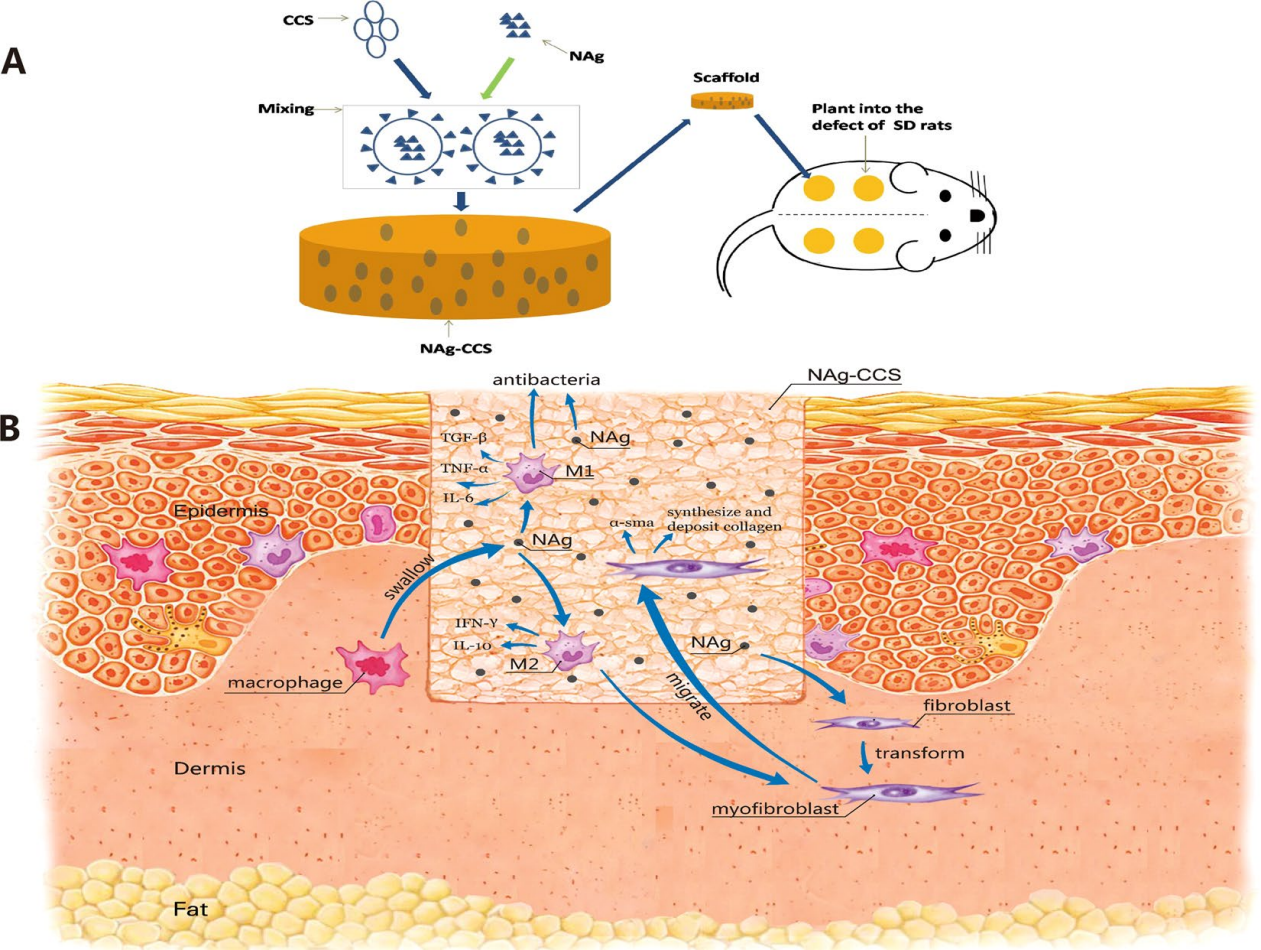
General view of our study. (**A**) Graphic illustration of NAg-CCS scaffold implanted into the defects of male SD rats. (**B**) The possible mechanisms of NAg accelerated cutaneous wound healing.

In this study, we developed a novel NAg-CCS by combining NAg and collagen-chitosan scaffold (CCS) (Fig. 1A), a dermal scaffold with good mechanical strength, flexibility, tear resistance and biocompatibility. *In vitro* and *in vivo* studies were conducted to evaluate the antimicrobial property of NAg-CCS and determine its role in wound healing. The mechanisms of action NAg are postulated to have were illustrated in Fig. 1B.

## Results

### NAg promotes fibroblast migration and myofibroblast differentiation

**Figure 2:**
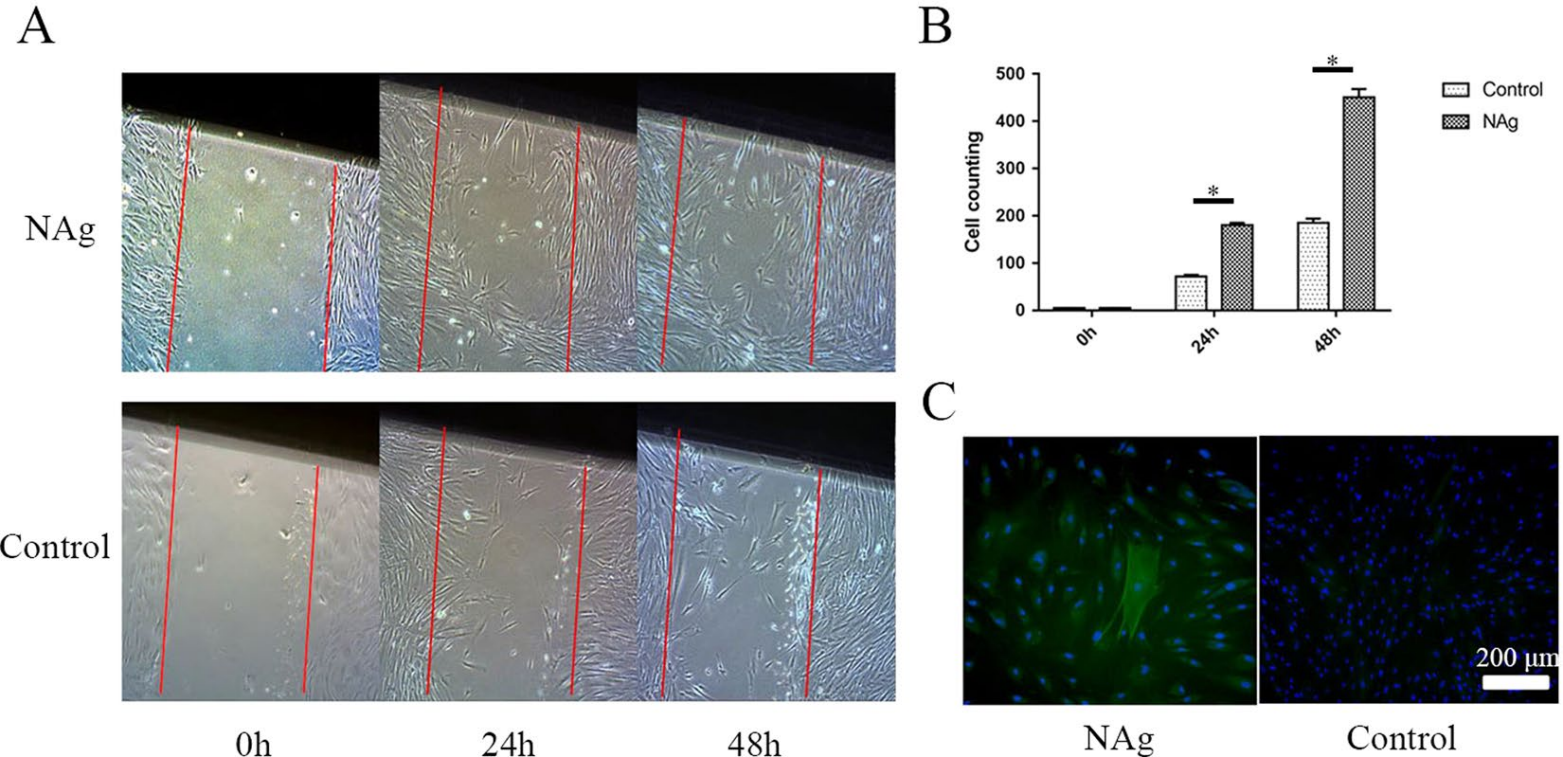
NAg accelerated migration fibroblast in scratch assay. (**A**) Scratch assay of fibroblast treated with NAg (10 ppm) for 0, 24, and 48 hours. (**B**) The relevant counting of cells in scratch area. *Indicates a statistically significant difference, P < 0.05. (**C**) Immunostaining of α-SMA in fibroblast. Left, cultured with NAg; right, control group. The white Bar indicates 200 μm.

**Figure 3:**
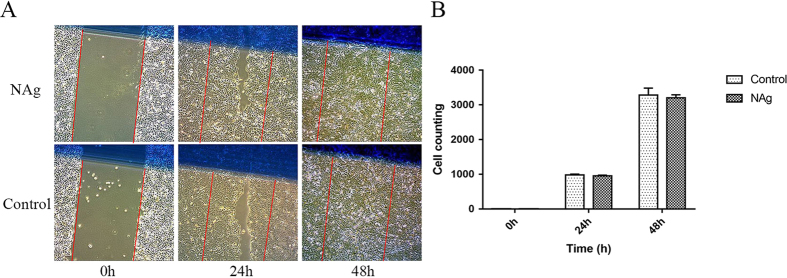
NAg did not prevent HaCaT cells from growth and migration. (**A**) Scratch assay of HaCaT treated with NAg for 0, 24, and 48 hours. (**B**) The relevant counting of cells in scratch area. *Indicates a statistically significant difference, *P* < 0.05.

In order to characterize the potential influence of NAg on various skin cell types individually, keratinocytes and fibroblasts were cultured. The extent of regrowth to close the scratch wound was measured after 0, 24 and 48 hours of incubation in medium containing NAg (10 ppm). Restoration of the full cellular density of the mesothelium in fibroblasts was faster in the NAg group than that in the control group. In other words, NAg promoted the migration rate of fibroblasts obviously at each time point (P < 0.0001, Fig. 2). In order to further confirm this finding, cells were stained for α-SMA, a marker of myofibroblasts. As shown in Fig. 2C, there was a marked increase in α-SMA expression in fibroblasts treated with NAg, demonstrating a transformation in cell phenotype from fibroblast to myofibroblast. No significant difference in migration abilities was found between the two groups of HaCaT cells, as displayed in Fig. 3.

### NAg-CCS and CCS share similar structural characteristics based on SEM observation

**Figure 4:**
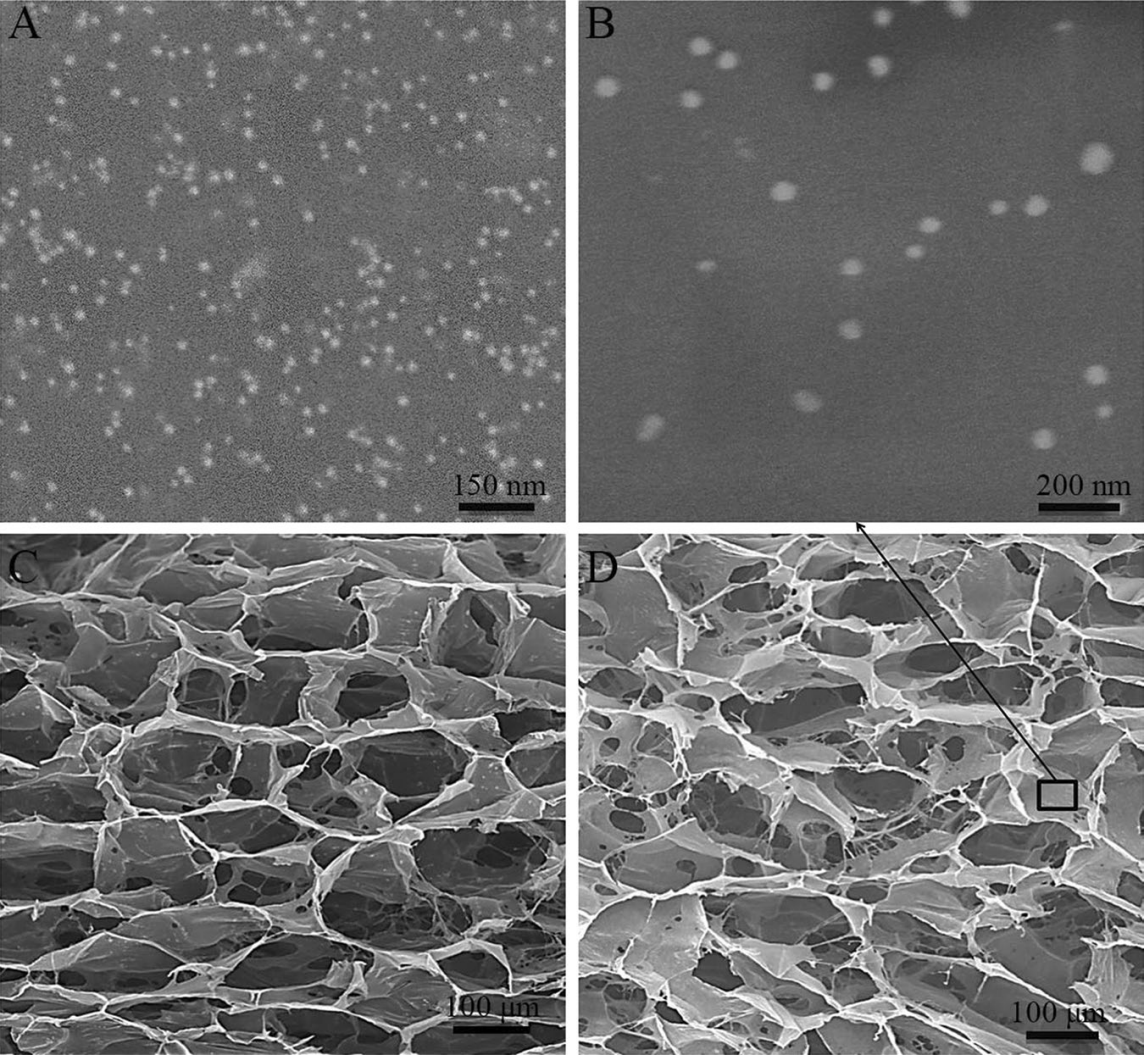
SEM images of NAg, CCS and NAg-CCS. (**A**) SEM images of NAg, (**B**) wrapped nanoparticles dispersed homogeneously in the CCS, (**C**) CCS, and (**D**) NAg-CCS.

The morphologies of NAg, CCS and NAg-CCS were shown in Fig. 4. The diameter of NAg used in this study ranged from 10 to 25 nm, as shown in Fig. 4A. Spherical NAg was randomly distributed in the scaffold, and the diameter of the encapsulated particles was 50–90 nm (Fig. 4B). Both CCS (Fig. 4C) and NAg-CCS (Fig. 4D) had a mean pore size of 136 ± 5 µm and a porosity of 93.6%, and there was no structural difference between NAg-CCS and CCS.

### NAg strengthens the antimicrobial activity of scafford *in vitro*

**Figure 5:**
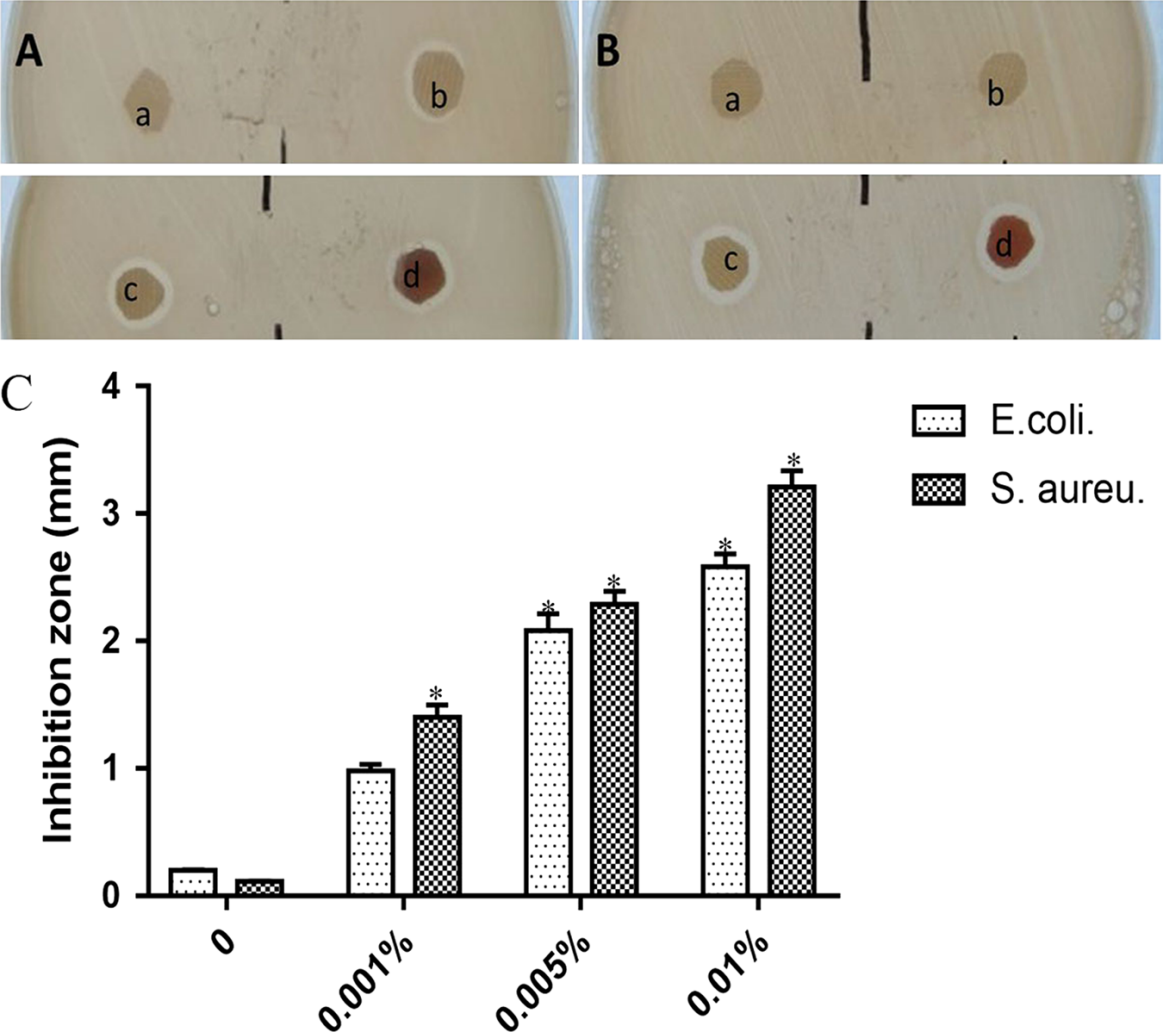
Antimicrobial studies of NAg-CCS against E.coli. and S. aureus. with diffenrent concentrations of NAg. Antimicrobial studies of NAg-CCS against *E.coli*. (**A**) and *S. Aureus*. (**B**). (a) CCS control, (b) CCS + 0.001% nanosilver, (c) CCS + 0.005% nanosilver and (d) CCS + 0.01% nanosilver. (**C**) The inhibition zone of different scafflods. *Indicates a statistically significant difference, *P* < 0.05.

An obvious difference was observed in antimicrobial activity between NAg-CCS and CCS groups (Fig. 5). More antimicrobial activity was observed in the NAg-CCS group than that in CCS group. Moreover, with the increase of NAg concentrations, antibacterial activity improved in both groups. The minimal inhibitory concentration of NAg was ≤ 10 ppm. Hence, we prepared the scaffolds for animal experiments with a NAg concentration of 10 ppm.

### NAg-CCS accelerates cutaneous wound healing *in vivo*

**Figure 6:**
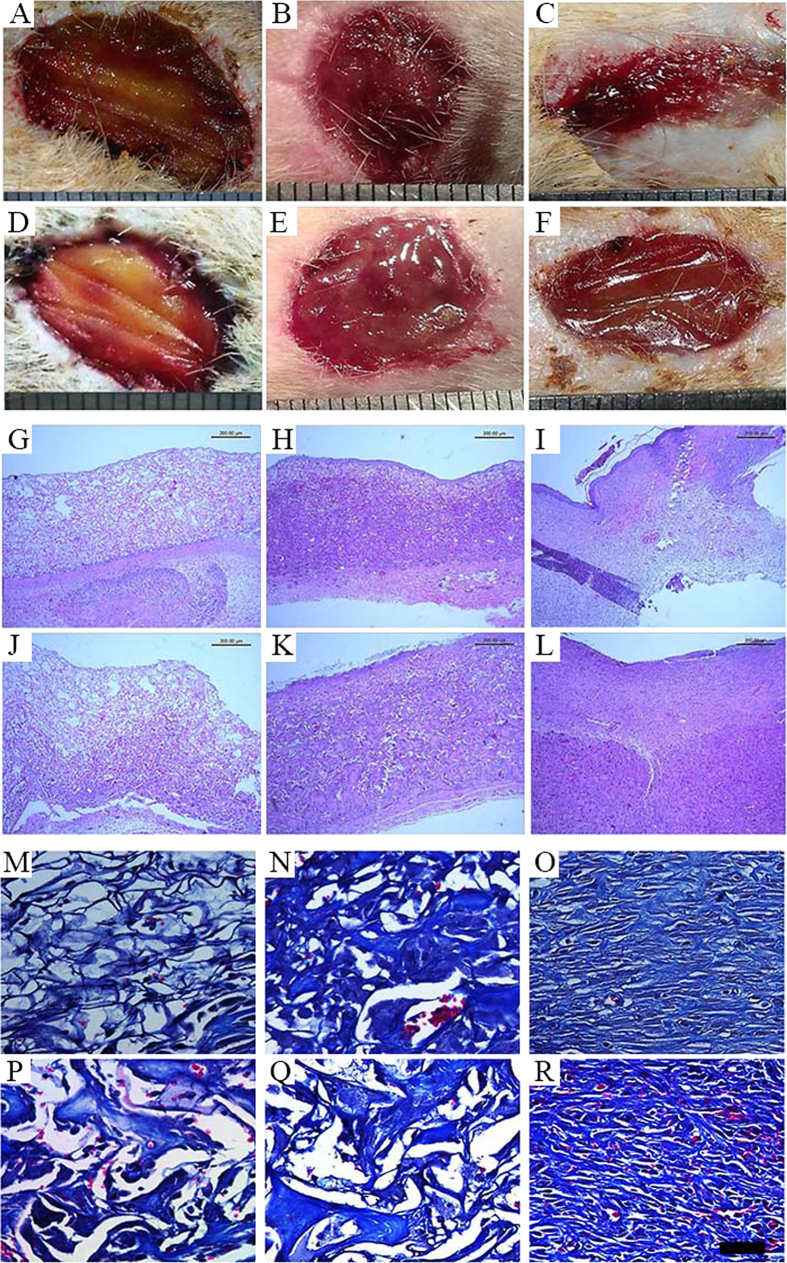
Gross and pathological observations of NAg-CCS (**A**–**C**) and CCS (**D**–**F**) at week 1, 2 and 4 post transplantation. Light blue indicates newly-formed collagen, and red indicates cytoplasma.

Photographs were taken to record and analyse the *in vivo* performance of the scaffolds after implantation. In the NAg-CCS group, no evident inflammatory reaction (infection, fistula, or fibrous capsule) was observed at either the implantation sites or adjacent sites for week 1, 2 and 4 post transplantation. However, in the CCS group, detachment of implanted scaffolds and obvious infection were frequently observed, as well as adherence of festering scaffolds to the wound. After no more than four weeks, wounds in the NAg-CCS group gradually narrowed and achieved earlier epithelialization (Fig. 6A–C) compared with the CCS group (Fig. 6D–F). Residual area of the transplanted wounds at each time point was also counted. At week 2, a smooth skin surface was observed with a residual area of 2.11 ± 0.23 cm^2^ in the NAg-CCS group; while the CCS group revealed a rough appearance with a size of 2.97 ± 0.13 cm^2^. At week 4, the average size of the residual area in the NAg-CCS group (1.66 ± 0.14 cm^2^) was significant smaller than that in the CCS group (2.80 ± 0.05 cm^2^).

Results of the H&E staining were shown in Fig. 6. Abundant fibroblasts and ECM were observed at the bottom of scaffolds in the NAg-CCS group (Fig. 6G–I), while granulation tissues could only be observed in the adjacent areas in the CCS group (Fig. 6J–L). Additionally, inflammatory cells were mainly distributed in the center of scaffolds in the CCS group while NAg-CCS was infiltrated with fewer inflammatory cells. At week 1, an obvious layer of epidermal cells formed on the surface of NAg-CCS with less inflammatory infiltration. At week 2, the implants were well integrated with adjacent tissue. The number of granulocytes increased in both groups, especially in the CCS group. After no more than 28 days, wounds in the NAg-CCS group were covered with epidermis. However, the unfailing inflammatory reaction hindered wound healing in the CCS group and no sign of epithelialization was observed within 4 weeks after implantation.

Masson staining demonstrated that new collagen in the NAg-CCS group was equally distributed in the scaffolds (Fig. 6M–O), while only bundles of old collagen accompanied by inflammatory cells could be observed in the CCS group(Fig. 6P–R). By day 28, the morphology and distribution of new collagen in the NAg-CCS group was similar to that in normal dermis.

### NAg-CCS attenuates inflammatory response through regulating macrophage activation

**Figure 7:**
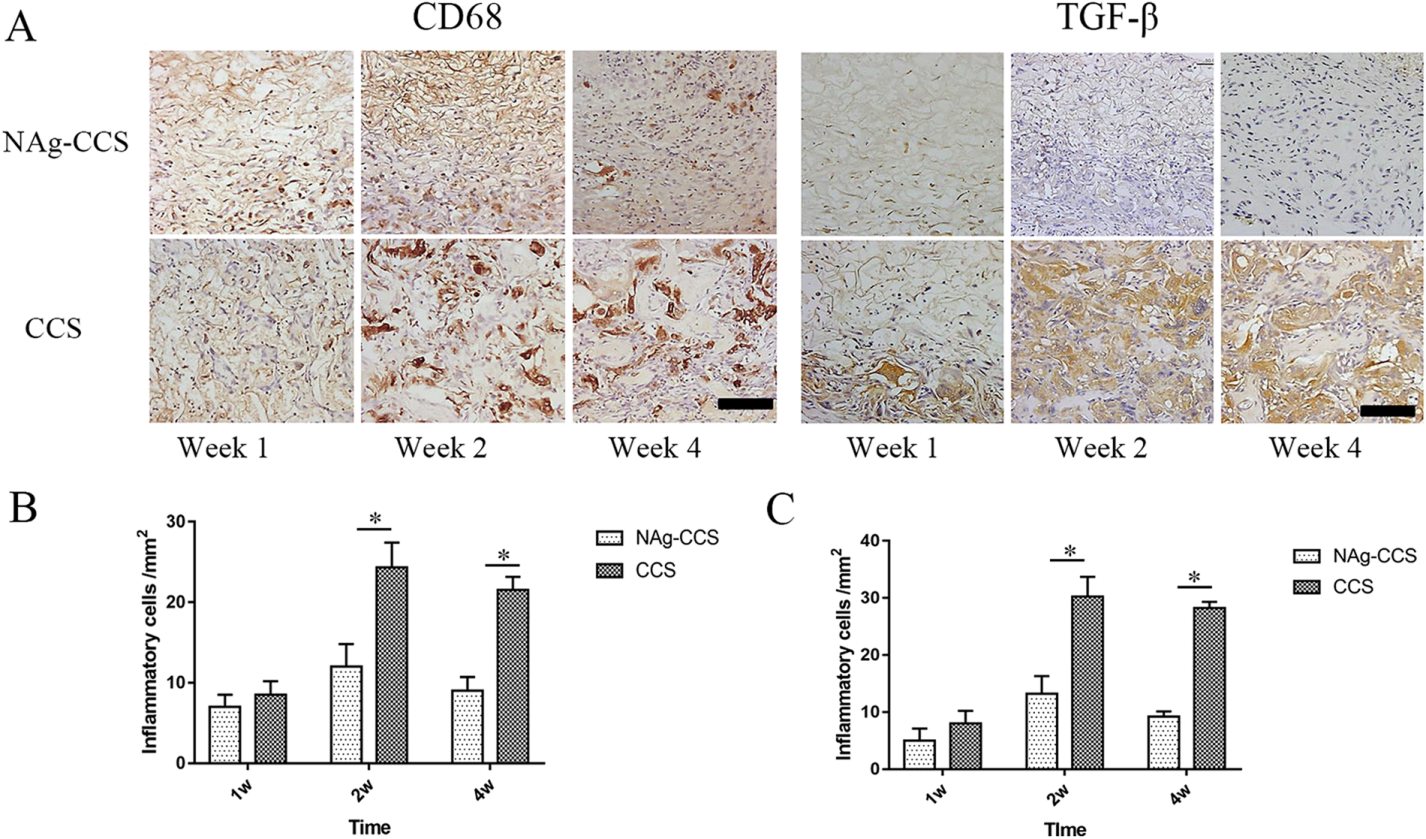
Immunostaining of CD68 and TGF-β of sections of NAg-CCS and CCS at week 1, 2 and 4 post transplantation. (**A**) CD68 and TGF-β immunohistochenmical staining and the relevant counting of inflammatory cell with positive staining of NAg-CCS (**B**) and CCS (C). *Indicates a statistically significant difference, *P* < 0.05. The bar indicates 50 μm.

In order to characterize the new inflammatory status of the implants, immunohistochemical staining for CD68 (a marker of macrophages) and TGF-β1 (a marker related to inflammation and scar-forming) was performed. At week 1, cells were abundantly distributed in adjacent tissues. At week 2, specifically stained cells appeared in the internal part of the implants and their density in the NAg-CCS was obviously lower than that in the CCS group (Fig. 7). At week 4, all groups displayed strong positive staining and fewer positive cells for TGF-β1 were observed in the NAg-CCS group compared with the CCS group. The expression of CD68 in the NAg-CCS group was significantly lower than that in the control group.

**Figure 8:**
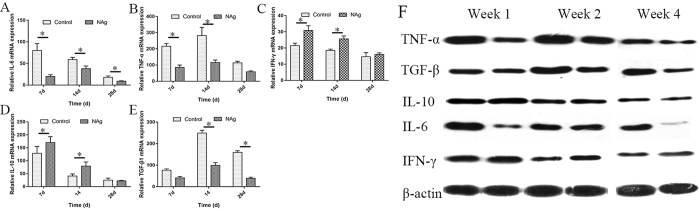
NAg-CCS downregulated inflammatory mediators and upregulated macrophage activation associated factors in full-thickness wounds at day 7, 14 and 28 post transplantation. Real-time quantitative analysis of TNF-α (**A**), IL-10 (**B**), IL-6 (**C**), IFN-γ (**D**) and TGF-β1 (**E**) mRNA expression. (**F**) Western blotting analysis of TNF-α, CD68, IL-10, IL-6 and IFN-γ of the NAg-CCS (left) and CCS (right) implanted at day 7, 14, 28 post transplantation, respectively. The gels/blots were cropped and full-length gels and blots were included in the Supplementary Information file.

To assess the anti-inflammatory properties of NAg, a quantitative evaluation of the expression of TNF-α, TGF-β, IL-10, IL-6 and IFN-γ was performed at both mRNA and protein levels. As indicated in Fig. 8A, IL-6 levels in the CCS group increased, with a prolonged implantation time for 4 days, followed by a decrease at day 7. In contrast, there was always a significantly lower IL-6 expression in the NAg-CCS group compared with the control group, although the expression increased on day 7 after surgery. Figure 8B showed that the mRNA expression of TNF-α constantly increased in the CCS group, but was significantly suppressed in the NAg-CCS group. TGF-β mRNA expression maintained at a stable and low level in the NAg-CCS group, while it significantly increased from day 7 and reached a peak at day 14 (Fig. 8E). These results were almost opposite to the variation tendency of IL-10 and IFN-γ mRNA (Fig. 8C and D), suggesting the inhibition of IL-6, TGF-β and TNF-α in the NAg-CCS group.

Western blot analysis of the tissue samples was further conducted to detect protein levels of TNF-α, TGF-β, IL-10, IL-6 and IFN-γ. As shown in Fig. 8F, each sample in the NAg-CCS group showed weaker bands for TNF-α, TGF-β and IL-6, when compared with the corresponding bands in the CCS group for week 1, 2 and 4 post transplantation, while the results for IL-10 and IFN-γ revealed stronger bands in the NAg-CCS group at each time point.

### NAg-CCS normalizes the process of cutaneous wounding healing

**Figure 9:**
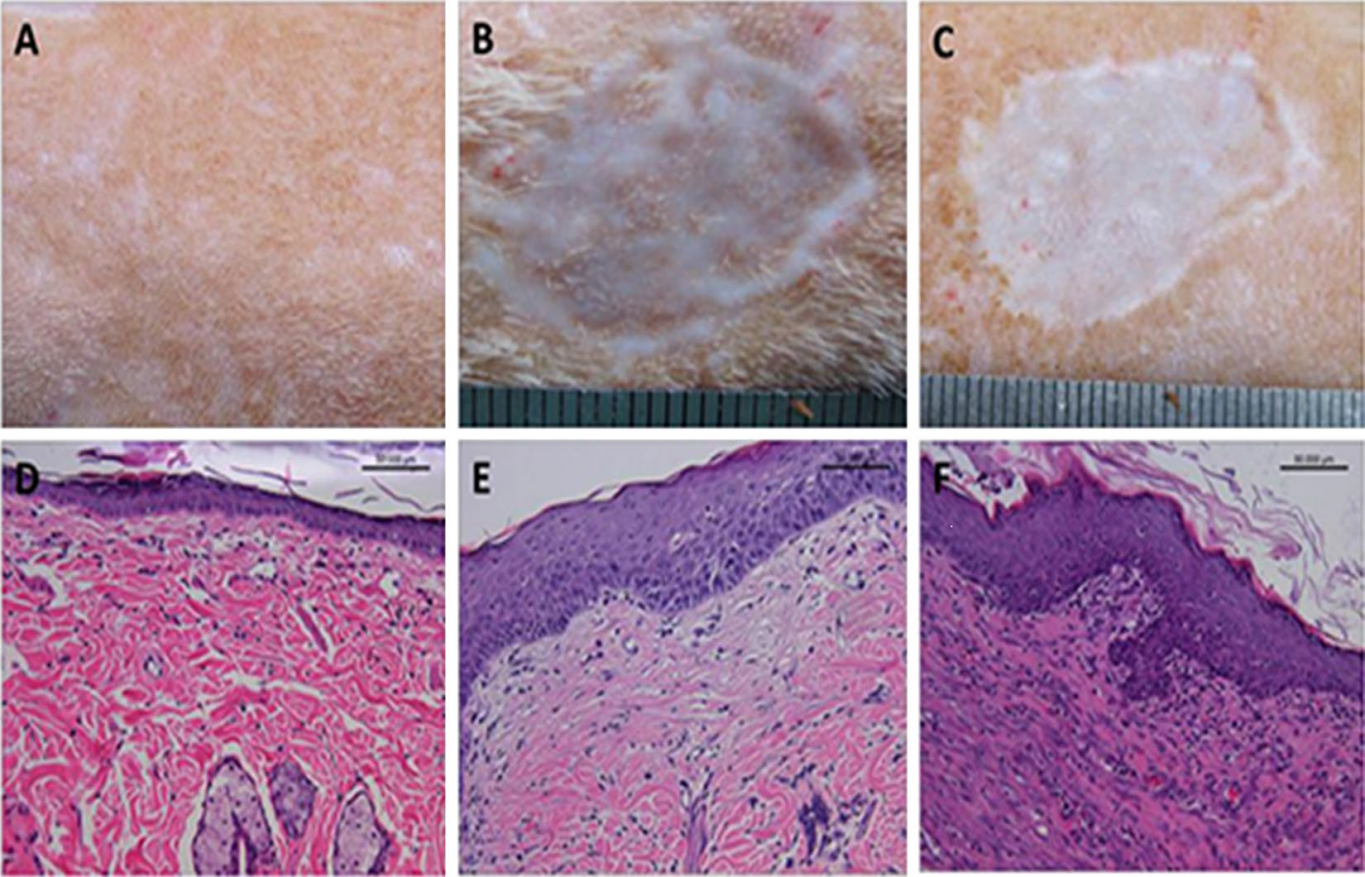
Gross views (**A**–**C**) and H&E staining of sections (**D**–**F**) of normal skin (**A**,**D**) and wounds treated by NAg-CCS (**B**,**E**) and CCS (**C**,**F**) at day 60 post transplantation, respectively. Ultra-thin autografts were transplanted at day 10. The bar indicates 50 μm.

For the sake of evaluating the long-term therapeutic outcomes, full-thickness skin wounds were treated with NAg-CCS (Fig. 9B and E) and CCS (Fig. 9C and F) respectively. Ultra-thin autografts were transplanted at day 10. Healed wounds in both groups were observed in gross view and sampled for H&E staining at day 60 post transplantation. It was shown in Fig. 9 that the regenerated skin by NAg-CCS had a similar structure to normal skin, while obvious scar tissue was found in control group.

## Discussion

Nanocrystalline silver dressings were commercially introduced as antimicrobial dressings in 1998. Recent advances in nanotechnology have resulted in the ability to produce pure silver as nanoparticles. When used in burn injury wounds, NAg could promote wound healing, which demonstrates that NAg could initially improve the healing of wounds on the basis of its antimicrobial property^16^. However, our *in vitro* study indicated that antimicrobial activity of NAg may not be the only factor that promoted wound healing. The scratch assay performed here was used as a *in vitro* model of wound healing. NAg at a concentration of 10 ppm could improve the migration rate of fibroblasts. Moreover, fibroblasts treated with NAg (10 ppm) also expressed higher levels of α-SMA (a marker of myofibroblasts), suggesting that fibroblasts treated with NAg had the potential of transforming into myofibroblasts. As myofibroblasts possess strong abilities of migration and contraction, NAg may play an important role in accelerating dermal regeneration and speeding wound repair by transforming fibroblasts into myofibroblasts. Therefore, we incorporated NAg into the scaffold CCS to improve the dermal regenerative properties of the scaffold itself. However, there was no significant difference in the migration rate of HaCaT between the NAg group and the control group. This could be due to the concentration of NAg (10 ppm) we used in the scratch assay and follow-up animal experiments did not reach the effective concentration for HaCaT cells. Both CCS and NAg-CCS had similar pore size, porosity and structural characterization. In addition, NAg was uniformly distributed on the surface of scaffold and inside the interconnected pores.

Inflammatory response and microbial environment are important components in wound healing. Many experiments have demonstrated that nanocrystalline silver dressings could improve wound healing both *in vivo* and *in vitro*, which may also result from the particles’ anti-inflammatory activity^17, 18^. It is widely accepted that normal wound healing occurs in three stages: inflammation, repair and remodeling. Throughout the process of wound healing, macrophages play a vital role since they have the ability to phagocytose and clear necrotic tissue, pathogenic microorganisms and foreign matter^19^. They can also secrete various cytokines and chemokines to stimulate cell proliferation and collagen deposition, promoting vascularization and granulation^20, 21^. There are two different activation patterns of macrophages, i.e. classical macrophage activation (M1), in which macrophages act as immune and inflammatory cells in the earlier phase of wound healing, and alternative macrophage activation (M2), presented as the “almighty” repairing cells and dominate at the later stage of wound healing^22, 23^. These finding demonstrates regular variations of different macrophage activation types in the normal wound healing process, which coincides with the regular pattern of wound healing. However, the unusual activation of macrophages of non-healing or infected wounds makes it difficult to reduce inflammation under pathological conditions. If an agent or drug could be used to avoid the unusual activation or infection, the healing process might reasonably continue. So we hypothesize that, as an antibacterial and anti-inflammatory agent^24^, NAg could regulate the wound’s abnormal activation of macrophages and improve the progress of inflammatory stages to accelerate wound healing.

The combination of excellent tissue engineering materials and nanoparticles was used to construct a dermal scaffold with antibiotic and anti-inflammation abilities. However, the simple physical absorption fabricating a NAg loaded scaffold often results in a robust release of the particles at an early stage. These particles may participate in antibiosis or be swallowed by pathogenic bacteria and M1 activated macrophages. The scaffolds improved the condition of the wound bed and ensured the progress of normal inflammatory stage without undesirable extension or aggravation, which would also avoid scar formation. After the stable inflammatory stage, repair of the wound begins with the activation of M2 macrophages. During this conversion, the scaffolds which contain NAg were a perfect choice for functionalized dermal substitutes on the surface, of which epithelial cells and other stem cells accumulated to trigger epithelialization in seven days. If this type of rapid epithelialization could be used for large area skin defects, it would be a major breakthrough in the field of wound repair and regeneration.

With the development of proteomics, scientists found that NAg can suppress the expression of pro-inflammatory cytokines, which may be partly due to the induction of apoptosis and suppression of MMP activity^25^. Nanocrystalline silver suppresses the production of many cytokines such as TNF-α, IL-8, TGF-β and IL-12, which reduces inflammation^26, 27^. NAg released into the blood circulation has been shown to be taken up by peripheral blood mononuclear cells, causing apoptosis and inhibiting the expression of IL-5 and TNF-α^28, 29^. In the current study, there were similar variations in inflammation-related cytokines, which were tested by immunohistochemical staining, RT-qPCR and western blot analysis. CD68, as the most reliable marker of macrophages, is expressed in macrophages, monocytes, Kupffer cells, osteoclasts, granulocytes and their precursors. In addition, CD68 is a direct indication of inflammatory cell infiltration. TGF-β and IL-6 have been shown to be potent stimulators of fibroblast proliferation, which is diminished in fetal wounds^30–32^. This finding indicates that the overexpression of these factors in wounds would lead to delayed and undesirable wound healing. The low expression of CD68, TGF-β and IL-6 provides evidence for decreasing inflammatory reaction and predicating wound healing with inhibited scars. However, not all cytokines were inhibited. RT-qPCR and western blot analysis results indicated that these pro-inflammatory factors (IL-6, TNF-α and TGF-β) were depressed, while anti-inflammatory factors (IFN-γ and IL-10) were enhanced. IFN-γ plays an important role in tissue remodeling of wounds by retarding collagen production and lattice cross-linking with an increase in collagenase production, in order to reduce wound contraction^29, 33^. IFN-γ is the inducer for the activation of M2 macrophages, which functions as pro-inflammation cells^33^. The inhibition of these cytokines may be one of the many mechanisms of NAg to regulate the inflammatory reaction in wounds. As IFN-γ has been demonstrated as a potent antagonist of fibrogenesis, it may play a role in the regulation of TGF-β1 production by inhibiting fibroblast proliferation and matrix production. The expression level of IL-10 was obviously higher in NAg-CCS than that in the control group, especially at four days after injury. When neutralizing antibodies against IL-10 were applied to incision wounds, an increase in the infiltration of neutrophils and macrophages, as well as the expression of chemokines and pro-inflammatory cytokines were observed^34, 35^. These findings gave us great confidence in the application of NAg for wound healing. However, it indicates a complicated mechanism for the immune response to NAg, which needs further investigation. Therefore, NAg, of a proper size and concentration, could be a powerful agent to prevent unusual activation or infections and maintain a reasonable balance of macrophage activation.

Nonetheless, the findings reported in this study not only confirm the efficient antimicrobial and anti-inflammatory properties of NAg, but also provided a clue in determining the mechanism of interaction between NAg and skin cells. An overall decrease in inflammatory response at the local wound sites was observed in SD rats in NAg-CCS group with better healing outcome, further confirming that inflammation is a prerequisite of wound healing, but extensive inflammation is detrimental in the long term. Combining NAg and a dermal substitute could not only provide a carrier for particles to preserve their *in situ* functions, but also empower the scaffolds with the ability to stimulate immune response and cell migration, and achieve a rapid regeneration and high-quality repair.

We conclude that NAg can modulate local inflammatory responses and promote fibroblast migration by interacting with macrophages and fibroblasts, in order to achieve transformation of the predominant cell types. However, few studies have reported the mechanism of the interaction between NAg and cells. Antibiosis exerted by NAg undoubtedly plays an important role in wound healing. More importantly in this case, they might have the ability to regulate behaviors of certain types of cells during the process. Further studies are needed to determine the underlying mechanisms.

## Methods

### Materials

The nanoparticles used in this study were NAg of 20–40 nm in diameter (Huzheng-NanoSilver), which were obtained from Huzheng Nano Technology Co., Ltd. (Shanghai, China). Chitosan (deacetylation degree: ≥85%, Mη: 1.06 × 10^5^–1.71 × 10^5^) was purchased from Sigma (USA). Type I collagen was extracted and purified from fresh bovine tendons by swelling, deleting telopeptides with pepsin (Sigma, USA), and performing sufficient dialysis, as previously described^4^. Analytical grade reagents and deionized water were utilized in these experiments.

### Cell culture

A mouse embryo fibroblast cell line and a spontaneously immortalized human skin keratinocyte line (HaCaT) were purchased from ATCC and cultured as described. Briefly, fibroblasts were seeded and grown in Dulbecco’s modified Eagle’s medium (DMEM; Sigma, USA) with 10% fetal bovine serum (FBS) and a penicillin/streptomycin mixture under an atmosphere of 5% CO2 at 37 °C. HaCaT cells were seeded and grown in RPMI-1640 medium (Sigma, USA) containing 10% FBS and a penicillin/streptomycin mixture under an atmosphere of 5% CO2 at 37 °C. The cultures were passaged twice before the experiments.

### Scratch assay

Fibroblast and HaCaT cells were grown in six-well plates to a confluent monolayer. The cell monolayer was scraped in a straight line with a 200-µL pipette tip. Cell debris was removed by washing the cells three times with phosphate-buffered saline (PBS). Then, cells were immediately cultured with NAg (10 ppm) and 0.2% FBS as control. The dishes were placed under a phase-contrast microscope, and the first image of the scratch was acquired. Cells were incubated at 37 °C for 48 hours, and observed under a phase-contrast microscope (matching the reference point) at 0, 24 and 48 hours.

In order to precisely count the number of cells in the scratch area, three culture dishes were prepared for each group at each time point. Haematoxylin was used to stain the nuclei, which were enumerated. All counting procedures were conducted separately by two pathologists.

### Immunofluorescence staining

For the immunostaining of α-SMA in the cell culture, fibroblasts were grown in chambers and mounted on glass slides with cover slips. Then, NAg was added to the medium with a final concentration of 10 ppm. Next, cells were incubated for 48 hours and fixed in 4% paraformaldehyde. Samples were washed with 0.25% Trion X-100 (Sigma), and bovine serum albumin (BSA, 1%) was used to block nonspecific binding for 1 hour. Sections were then washed with PBS before the addition of anti-α-SMA antibody (Abcam Ltd., USA; 1:200). TRITC-conjugated goat anti-mouse anti-body (1:200) and TRITC-conjugated goat anti-rabbit antibody (1:200) were used as secondary antibodies, respectively. The samples were washed three times and stained with DAPI for 2 minutes before analysis by fluorescence microscope.

### Preparation of NAg-CCS

Bovine type-Ι collagen and chitosan at a mass ratio of 9:1 were dissolved in 0.5 M of acetic acid solution to form a 0.5% (w/v) solution, and was kept at 4 °C until use. The nanosilver solution was added to the collagen/chitosan hybrid scaffold (CCS) hydrogel to achieve different concentrations (0, 2, 5, 10 and 20 ppm) of the NAg-CCS solution. In total, 3.2 ml of NAg-CCS was carefully poured on a flat plate (homemade model, 4.0 × 4.0 × 0.2 cm). The composite was incubated at 4 °C for 24 hours, frozen at −25 °C for three hours, and lyophilized for 24 hours to produce a hybrid scaffold. The porous CCS without the nanosilver was prepared and treated as a control, as previously described. All samples of CCS and NAg-CCS were cross-linked with EDAC/NHS, as previously described^7^. Before implantation in the animal experiment, the scaffolds were irradiated with a γ-ray (25 KGy) for 24 hours.

### Scanning electron microscopy (SEM) observation

After sectioning with a razor blade and coating with platinum, the morphologies of the nanosilver, NAg-CCS and CCS were observed using SEM (Philips XL30, Eindhoven, Netherlands). The accelerating voltage was set at 1.0 kV. In order to determine the mean pore size of the scaffolds and diameter of the particles, images of the NAg, CS and PMCS (cross-sectioned surfaces) were analysed. Three SEM images of each sample were taken randomly. In each image, ten apparent pores or particles were selected and measured with a ruler. The long and minor axes of pores or particles were measured in a perpendicular direction, and the average was taken as the mean size.

### *In vitro* antimicrobial activity

*E. coli* and *Staphylococcus aureus* were used to test the antibacterial potential of the composite scaffolds. Antibacterial activity was determined by the Kirby-Bauer disc diffusion method. The bacteria were grown overnight in tryptic soy broth. The disc diffusion method was performed on Mueller-Hinton agar (Himedia, India), and the zone of the inhibition was measured after 24 hour of incubation.

### Rat skin defect model

The experiments on rat skin defect model was performed according to protocols approved by the Ministry of Science and Technology of China and the Committee on Animal Care and Use of the Second Affiliated Hospital, School of Medicine, Zhejiang University, and strictly followed the National Institutes of Health Guidelines for the Care and Use of Laboratory Animals. Thirty-six male Sprague–Dawley (SD) rats (two months old, 250 ± 12 g) were purchased from the Experimental Animal Center of Zhejiang University. Prior to operations, anaesthesia was administered intraperitoneally with 3% pentobarbital sodium solution (Sigma) at a dose of 1.0 ml kg^−1^. An additional dose (0.5 ml kg^−1^) was administered, as required, to maintain deep anaesthesia. The removal of the back hair of the rat was performed with 8% Na2S aqueous solution. Four standardized full-thickness skin wounds (diameter = 2.0 cm) were excised from the dorsum of each rat with a distance of no less than 2.0 cm from the adjoining wounds. The NAg-CCS and CCS were planted into the defect. Then, the overlying dressing and fascia were closed with sutures to secure the implants in place. Following surgery, the animals were housed in separate cages and allowed to eat and drink *ad libitum*. Animals were monitored daily, but no antibiotic was administered. Finally, at 7, 14 and 28 days after the operation, the rats were sacrificed in batches. Following imaging of the wound sites, tissue specimens were harvested and cut apart along a uniform direction, and maintained in a 10% buffered formalin, liquid nitrogen and physiological saline solution for histological investigation and molecular biology analysis, respectively. A total of 36 rats underwent such operations, and six rats were assigned for each time point. At each time point, six rats were taken for histological investigation and six rats were taken for molecular biology analysis.

### Histology

For histological analysis, the samples were fixed in 4% formaldehyde/PBS solution, dehydrated with a graded series of ethanol, embedded in paraffin, and sectioned. Haematoxylin and eosin (H&E) staining and Masson’s trichrome staining were performed, and biopsies were observed under an optical microscope.

### Immunohistochemical analysis

For the immunohistochemical analysis, the sections were deparaffinized, washed with PBS three times, and blocked with 5% serum for 30 minutes. Then, the slides were treated with rabbit anti-CD68 primary antibody (1:100; Santa Cruz, USA) and rabbit anti-TGF-β primary antibody (1:100; Abcam, UK) at 4 °C overnight. The slides were further incubated with goat-anti-rabbit secondary antibody (1:200; Dako, CA, USA) at 37 °C for 30 minutes, developed with 3,30-diaminobenzidine tetrahydrochloride (DAB) solution, and counterstained with hematoxylin. Brown color indicates positive staining under an optical microscope.

### RNA isolation and RT-qPCR analysis

After 1, 2 and 4 weeks of implantation, the samples were harvested to isolate RNA for RT-qPCR analysis. Each sample was dissolved in 1 ml of TRIzol reagent (Invitrogen, USA), and the RNA was isolated. The content and purity of the RNA were measured with an ultraviolet spectrophotometer after dissolving RNA in DEPC–H2O solution. The generation of a cDNA library from the RNA template was performed using an M-MLV Reverse Transcriptase cDNA Synthesis Kit (Promega, USA). RT-qPCR was used to amplify TNF-α, IL-6, IL-10 and IFN-γ calibrator reference genes to glyceraldehyde 3-phosphate dehydrogenase (GAPDH). The reaction was performed with a iQTM5 Multiple Real Time Fluorescent Quantitation PCR Determinator (Bio-Rad, USA) for 40 cycles. The expression level of each target gene was normalized to GAPDH and calculated using the Bio-Rad iCycler software (version 2.0).

Fluorescent primers were devised using Primer Express 2.0 (ABI, USA) and Beacon Designer (Bio-Rad) software, which were obtained from Shanghai Bioengineering Co., Ltd. (China).

### Western blot analysis

Western blot analysis was performed as previously described^1^. Briefly, lysates extracted from frozen tissue specimens in RIPA lysis buffer (50 mM of Tris, 150 mM of sodium chloride, 0.1% SDS, 0.5% sodium deoxycholate, and 0.5% Triton X-100) were centrifuged at 12,000 rpm for 15 minutes at 4 °C, separated by SDS-PAGE, and transferred onto PVDF membranes (Millipore, USA). After incubation with antibodies overnight at room temperature, proteins were detected using an ECL Western Blotting Substrate (Pierce, USA) following treatment with 5% skim milk for one hour at room temperature. The antibodies used included rabbit anti-CD68 primary antibody (Abcam), rabbit anti-IL-8 primary antibody (Abcam), mouse anti-IL-6 primary antibody (Abcam), and mouse anti-IL-1β primary antibody (Abcam).

### Statistical analysis

All quantitative data were analysed with SPSS ver. 16.0 (USA), and were presented as mean ± standard deviation. Significant differences between specimens were evaluated using ANOVA. A *P*-value < 0.05 was considered statistically significant.

### Data Availability

The datasets generated during and/or analysed during the current study are available from the corresponding author on reasonable request.

## Electronic supplementary material

supplementary information
