# Supplementary material for: Silver nanoparticle loaded collagen/chitosan scaffolds promote wound healing via regulating fibroblast migration and macrophage activation
Source: Sci Rep. 2017 Sep 5;7:10489. doi: 10.1038/s41598-017-10481-0 (PMC5585259; doi:10.1038/s41598-017-10481-0)
Supplement: Supplementary file 1 — supplementary information [file 41598_2017_10481_MOESM1_ESM.pdf]

# Silver nanoparticle loaded collagen/chitosan scaffolds promote wound healing via regulating fibroblast migration and macrophage activation

Chuangang You[1,+], Qiong Li[1,+], Xingang Wang[1], Pan Wu[1], Jon Kee Ho[1], Ronghua Jin[1], Liping Zhang[1], Huawei Shao[1,*], Chunmao Han[1,*]

[1]Department of Burns & Wound Care Center, Second Affiliated Hospital of Medical College, Zhejiang University, Hangzhou, 310009, China

[*] Corresponding authors.

Address: Department of Burns & Wound Care Center, Second Affiliated Hospital of Medical College, Zhejiang University, Hangzhou, 310009, China. Tel: +86 571 87767187;

Email: 64207302@qq.com (H. Shao ); hanchunmao1@126.com (C. Han)

[+] These authors contributed equally to this work.

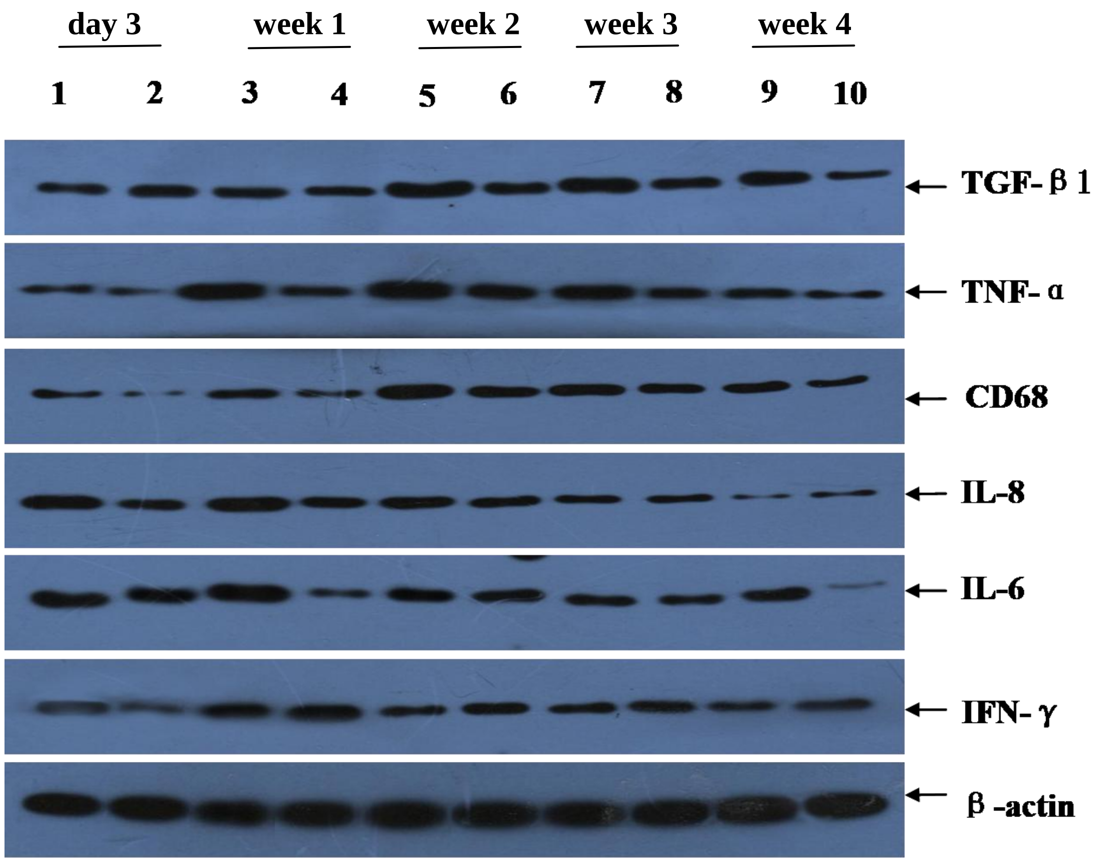


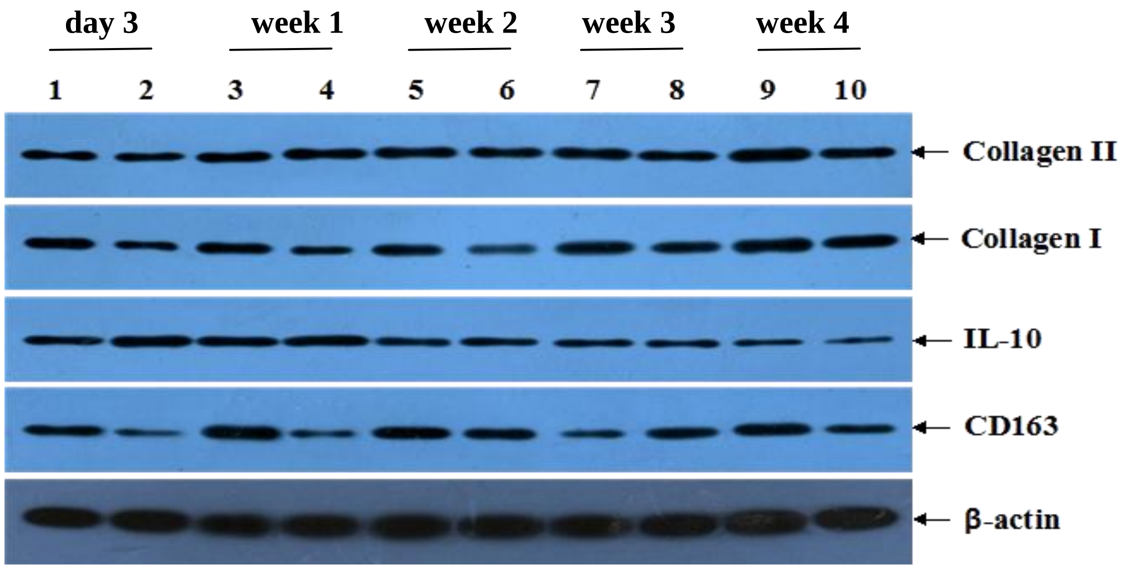


**Figure legends:**

Western blotting analysis of inflammatory mediators and macrophage activation associated factors of the NAg-CCS (left) and CCS (right) implanted at day 3, 7, 14, 21, 28 post transplantation, respectively. These full-length gels and blots were cropped and demonstrated in Figure 7.
